# Electrical impedance tomography for non-invasive identification of fatty liver infiltrate in overweight individuals

**DOI:** 10.1038/s41598-021-99132-z

**Published:** 2021-10-06

**Authors:** Chih-Chiang Chang, Zi-Yu Huang, Shu-Fu Shih, Yuan Luo, Arthur Ko, Qingyu Cui, Jennifer Sumner, Susana Cavallero, Swarna Das, Wei Gao, Janet Sinsheimer, Alex Bui, Jonathan P. Jacobs, Päivi Pajukanta, Holden Wu, Yu-Chong Tai, Zhaoping Li, Tzung K. Hsiai

**Affiliations:** 1 Department of Bioengineering, UCLA, Los Angeles, CA USA; 2 Department of Medical Engineering, California Institute of Technology, Pasadena, CA USA; 3 Department of Radiological Sciences, David Geffen School of Medicine at UCLA, Los Angeles, CA USA; 4 Department of Medicine, David Geffen School of Medicine at UCLA, Los Angeles, CA USA; 5 Department of Psychology, College of Life Sciences, UCLA, Los Angeles, CA USA; 6 Department of Biostatistics, Fielding School of Public Health, UCLA, Los Angeles, CA USA; 7 Department of Human Genetics, David Geffen School of Medicine at UCLA, Los Angeles, CA USA; 8 Computational Medicine, David Geffen School of Medicine at UCLA, Los Angeles, CA USA; 9 Division of Digestive Diseases, David Geffen School of Medicine at UCLA, Los Angeles, CA USA; 10 Greater Los Angeles VA Healthcare System, Los Angeles, CA USA; 11 Institute for Precision Health, David Geffen School of Medicine at UCLA, Los Angeles, CA USA; 12 Center for Human Nutrition, David Geffen School of Medicine at UCLA, Los Angeles, CA USA

**Keywords:** Biomedical engineering, Engineering

## Abstract

Non-alcoholic fatty liver disease (NAFLD) is one of the most common causes of cardiometabolic diseases in overweight individuals. While liver biopsy is the current gold standard to diagnose NAFLD and magnetic resonance imaging (MRI) is a non-invasive alternative still under clinical trials, the former is invasive and the latter costly. We demonstrate electrical impedance tomography (EIT) as a portable method for detecting fatty infiltrate. We enrolled 19 overweight subjects to undergo liver MRI scans, followed by EIT measurements. The MRI images provided the a priori knowledge of the liver boundary conditions for EIT reconstruction, and the multi-echo MRI data quantified liver proton-density fat fraction (PDFF%) to validate fat infiltrate. Using the EIT electrode belts, we circumferentially injected pairwise current to the upper abdomen, followed by acquiring the resulting surface-voltage to reconstruct the liver conductivity. Pearson’s correlation analyses compared EIT conductivity or MRI PDFF with body mass index, age, waist circumference, height, and weight variables. We reveal that the correlation between liver EIT conductivity or MRI PDFF with demographics is statistically insignificant, whereas liver EIT conductivity is inversely correlated with MRI PDFF (*R* = −0.69, *p* = 0.003, n = 16). As a pilot study, EIT conductivity provides a portable method for operator-independent and cost-effective detection of hepatic steatosis.

## Introduction

Obesity is the major risk factor associated with the development of nonalcoholic fatty liver disease (NAFLD), affecting more than a third of American adults, and the prevalence of severe obesity (BMI ≥ 35 kg m^−2^) is continuing to rise nationwide^1^. NAFLD is now one of the most common causes of cirrhosis requiring liver transplantation in the Western world^2,3^. A clinical challenge in the management of NAFLD resides in non-invasively detecting fatty liver (i.e., simple hepatic steatosis) at an early stage for intervention and monitoring its progression to steatohepatitis (hepatic inflammation), fibrosis (liver scarring), and ultimately cirrhosis^4,5^. While liver biopsy remains the gold standard for diagnosis of NAFLD, it carries substantial risks, including bleeding and is confounded by sampling bias and inter-observer variability^6^. While liver MRI proton-density fat fraction (PDFF) is recognized as the non-invasive reference standard for validating liver fat infiltrate^7,8^, it is cost-prohibitive for underserved communities and requires access to a scanner. Ultrasound elastography is also non-invasive, however, it is operator-dependent and resolution is limited ^9,10^. Thus, there remains an unmet clinical need to develop a cost-effective and portable method for early and operator-independent detection of fatty liver disease.

We have previously established the theoretical and experimental basis of electrical impedance tomography (EIT) for measuring liver fat content in the New Zealand White Rabbit model of fatty liver disease^11^. By virtue of tissue-specific electrical conductivity, fatty infiltrate in the liver is characterized by its frequency-dependent electrical impedance (Z) in response to applied alternating current (AC)^11^. At low frequencies, the lipid-bilayers impede the current flow, resulting in high conductivity, whereas, at high frequency, the bilayers serve as imperfect capacitors, resulting in tissue- and fluid-dependent impedance. This impedimetric property provides the basis for applying the multi-electrode array to measure tissue-specific conductivity, morphology, and volume^12–15^.For this reason, a body of literature has demonstrated brain EIT for functional studies, cardiac EIT for stroke volume, and transthoracic impedance pneumography for respiratory ventilation^16–19^.

In this context, we applied a portable multi-electrode belt to perform pairwise injection of alternating current (AC) to the liver in the upper abdomen, and we recorded the corresponding surface voltage to reconstruct the conductivity distribution for liver EIT ^11^. Specifically, we performed EIT voltage measurements by injecting electrical currents from 1–4 mA at 50 and 250 kHz. The current penetrated the abdomen to varying depths, and the resulting surface voltage was acquired by the multi-electrode array. Due to the varying free ion content, muscle and blood are more conductive than fat, bone, or lung tissue^12,20^. Fat-free tissue such as skeletal muscle carries high water (~ 73%), ions and proteins content, allowing for efficient electrical conductivity (S·m^−1^), whereas fat-infiltrated tissue such as fatty liver is anhydrous (steatosis)^21^, resulting in a reduction in conductivity^22^. This impedimetric property provides the basis to apply a liver EIT for the identification of fatty liver infiltrate. Unlike EIT for cardiopulmonary function focusing on the differential conductivity^12–19^, we solved the non-linear problem to reconstruct the absolute liver conductivity.

As a pilot study, we recruited overweight subjects (BMI > 25) to undergo liver 3 T MRI scans, followed by the portable EIT belt measurements. MRIs were acquired to provide the a priori knowledge of the liver boundary condition to solve the inverse problem for EIT reconstruction. We further compared and validated the subject-specific EIT conductivity with the liver MRI proton-density fat fraction (PDFF) as a reference standard for fatty liver infiltrate^23^. Next, we performed Pearson’s correlation analyses between the liver EIT or MRI PDFF with different parameters, including BMI (kg·m^−2^), age (years), waist circumference (cm), height (cm), and weight (kg). Following Bonferroni correction for multi-testing, correlation analyses revealed that neither EIT conductivity (S·m^−1^) nor MRI PDFF was correlated with these variables; but the liver EIT conductivity was inversely correlated with MRI PDFF. This inverse correlation holds promises for developing non-invasive and portable liver EIT for early detection of fatty liver content in the overweight individuals.

## Results

### Schematic workflow to compare and validate EIT reconstruction with MRI

**Figure 1:**
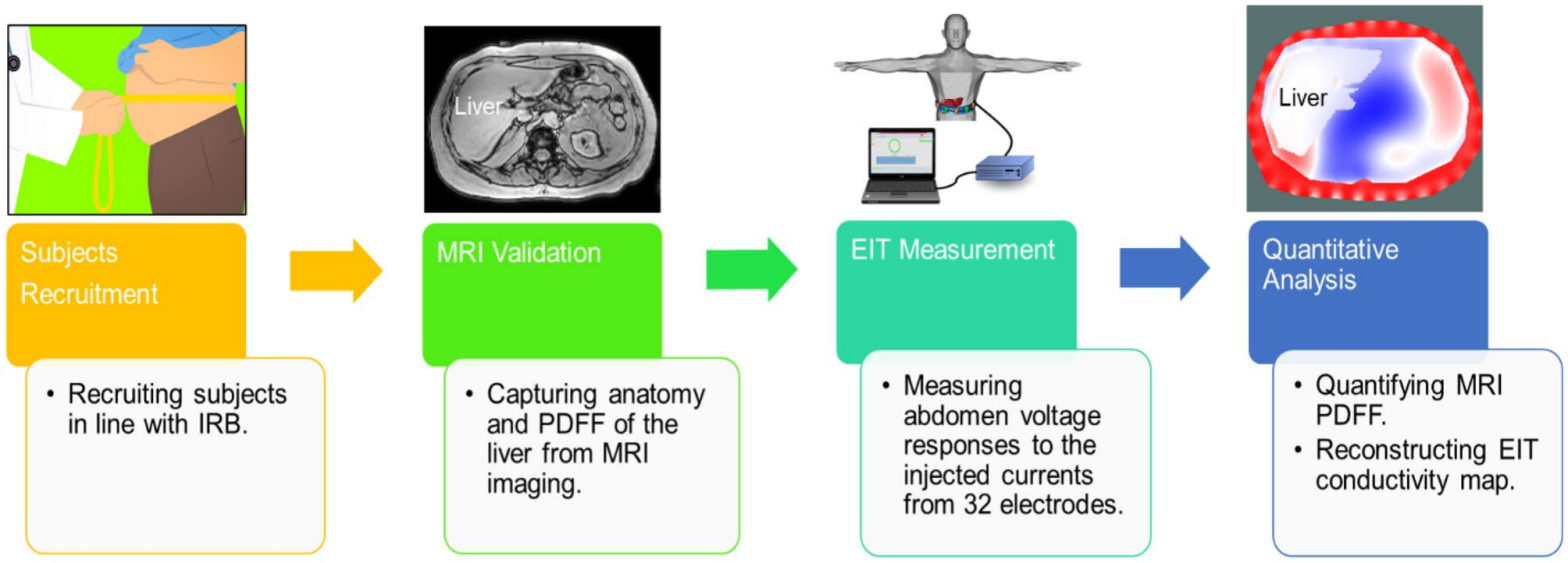
Schematic workflow of the comparison and validation of the MRI and EIT. Volunteers were recruited in line with the UCLA Institutional Human Subjects Protection Committee. Multi-echo MRI scans were performed to provide the liver anatomy and proton density fat fraction (PDFF), followed by the EIT measurements. Finally, the EIT conductivity maps were reconstructed and the MRI PDFF was used to quantify fatty liver infiltrate and to compare with EIT liver conductivity.

The subject recruitment complied with the guidelines of the UCLA Human Subjects Protection Committee, as described in the method section. The workflow and schematic setup (Fig. 1) depicted the individual subjects undergoing the liver MRI scans and MRI multi-echo imaging to acquire the PDFF, followed by the EIT measurements and reconstructions. The average liver MRI PDFF and the absolute EIT conductivity were quantified for the correlation analyses.

### Comparison between MRI multi-echo imaging and EIT images

**Figure 2:**
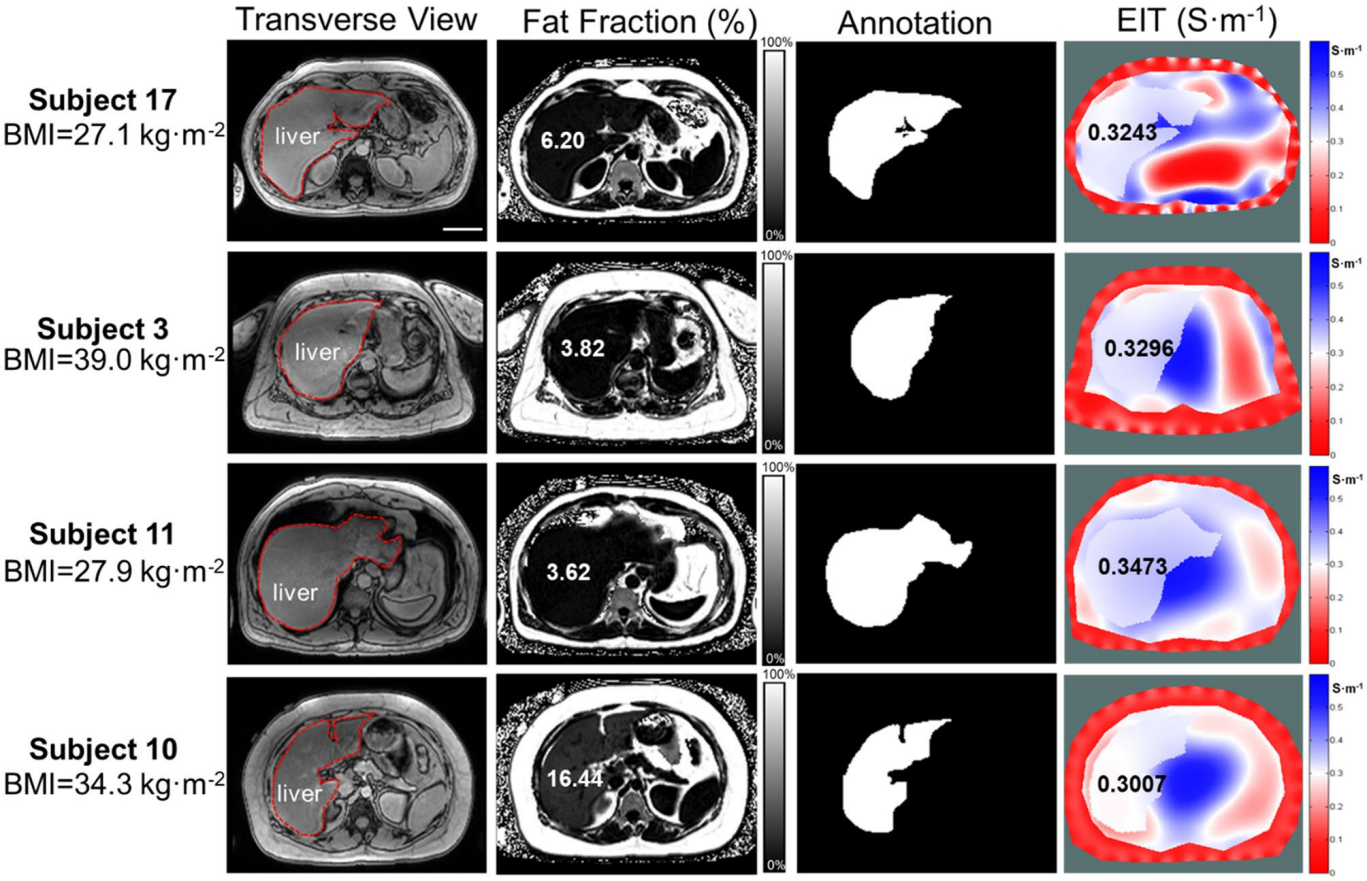
Representative MRI multi-echo and EIT images. Four representative subjects with different BMI values (Kg· m^−2^) were compared with MRI PDFF (%) and EIT conductivity (S·m^−1^), respectively. The transverse MRI views demarcate the liver anatomy, the fat fractions provide the corresponding MRI PDFF, annotation reveals the liver boundary condition following image segmentation, and 2-D EIT images unveil the abdomen conductivity distribution and average liver conductivity. The subject 17 with a BMI of 27.1 kg·m^−2^ developed a relatively high MRI PDFF (6.2%) and a low EIT liver conductivity (0.3243 S·m^−1^); whereas the subject 3 with BMI of 39 kg·m^−2^ developed a relatively low MRI PDFF (3.82%) and high EIT liver conductivity (0.3296 S·m^−1^). However, subject 11 with a BMI of 27.9 kg·m^−2^ developed a relatively low MRI PDFF (3.62%) in association with a relatively high EIT liver conductivity (0.3473 S·m^−1^), and the subject 10 with a BMI of 34.3 kg·m^−2^ also developed a relatively high MRI PDFF (16.44%) in association with a low EIT liver conductivity (0.3007 S·m^−1^). These initial comparisons suggest inconsistent correlations between BMI and MRI PDFF and EIT liver conductivity. Scale bar: 8 cm.

Liver MRI images provide the a priori geometric knowledge to reconstruct 2-D EIT images. This information includes the boundary conditions for: (1) the abdominal cross-section, (2) the peripheral tissues consisting of the skin, subcutaneous fat, and the ribs, and (3) the liver in the upper abdomen. For each subject, EIT liver conductivity and MRI PDFF were compared with the corresponding BMI value (Table 1). Furthermore, the representative abdomen MRI images for liver anatomy and PDFF, liver segmentation (annotation), and liver EIT conductivity distribution (S·m^−1^) were compared (Fig. 2). There was no significant correlation between the MRI PDFF and BMI or liver EIT and BMI. Subject 17 with a relatively lower BMI (BMI = 27.1 kg· m^−2^, PDFF = 6.2%, EIT = 0.3243 S·m^−1^) had a higher MRI PDFF than that of Subject 3 with a much higher BMI (BMI = 39.0 kg·m^−2^, PDFF = 3.82%, EIT = 0.3296 S·m^−1^). However, subject 11 with a low BMI value (BMI = 27.9 kg·m^−2^, MRI PDFF = 3.62%, EIT = 0.3473 S·m^−1^) had a lower MRI PDFF than that of Subject 10 with a higher BMI (BMI = 34.3 kg·m^−2^, MRI PDFF = 16.44%, EIT = 0.3007 S·m^−1^). Furthermore, despite similar BMI (27.1 kg· m^−2^ vs. 27.9 kg·m^−2^), the MRI PDFF of Subject 17 was around two times higher than that of Subject 11 (6.20 vs. 3.62%). Notably, the MRI PDFF for Subject 10 (BMI = 34.3 kg·m^−2^) was 4 times higher than that of Subject 3 (BMI = 39.0 kg m^−2^). These inconsistent relations support the notion that BMI is an inaccurate index to predict the levels of fatty liver infiltrate in the overweight individuals.

**Table 1:** BMI (Kg·m^−2^), MRI PDFF (%), EIT liver conductivity (S·m^−1^) and injection current of all subjects. (Subject 4: electrode malfunction, Subject 14: renal failure, Subject 18: leukemia, * asterisk).

Subjects	BMI(Kg·m^−2^)	EIT (S·m^−1^)	MRI PDFF (%)	Injection current (mA)
1	34.4	0.3518 ± 0.0192	2.14	1
2	49.7	0.3290 ± 0.0122	4.05	1
3	39.0	0.3296 ± 0.0130	3.82	2
4*	33.0	0.3819 ± 0.0224	27.89	3
5	30.6	0.3377 ± 0.0211	10.51	2
6	36.3	0.3444 ± 0.0322	4.14	2
7	29.3	0.3280 ± 0.0288	2.41	3
8	37.8	0.3405 ± 0.0134	2.25	2
9	32.0	0.3381 ± 0.0170	6.53	2
10	34.3	0.3007 ± 0.0167	16.44	2
11	27.9	0.3473 ± 0.0168	3.62	2
12	46.8	0.3307 ± 0.0113	5.14	2
13	38.9	0.3305 ± 0.0160	3.31	3
14*	25.5	0.3010 ± 0.0160	2.11	2
15	33.7	0.3306 ± 0.0127	10.78	2
16	27.4	0.3407 ± 0.0267	1.08	3
17	27.1	0.3243 ± 0.0125	6.20	3
18*	46.9	0.3455 ± 0.0149	18.56	2
19	29.8	0.3507 ± 0.0189	2.29	2

### EIT conductivity versus MRI PDFF

**Figure 3:**
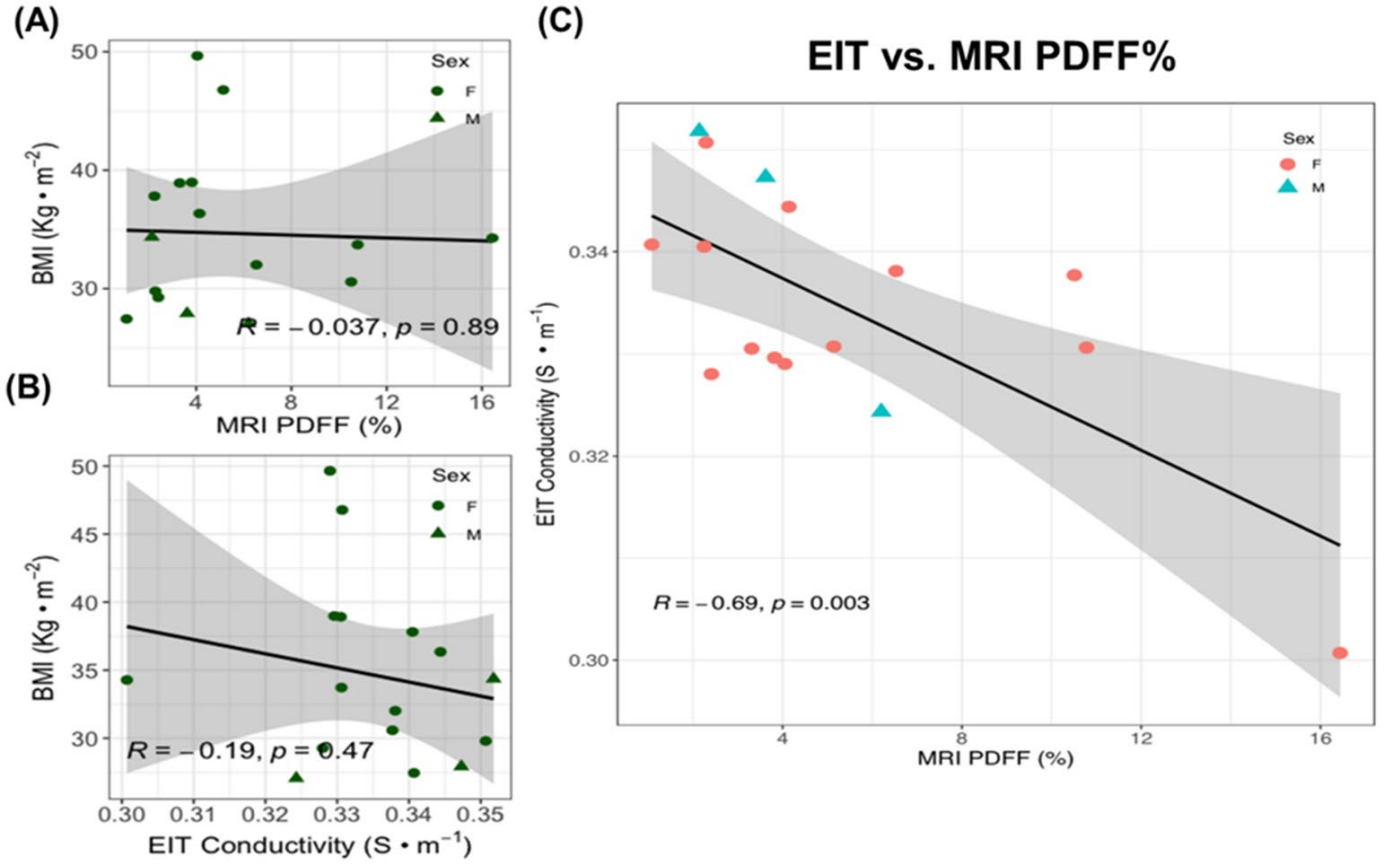
Statistical analyses of BMI vs. MRI PDFF and vs. EIT liver conductivity. (**A**) BMI values are not significantly correlated with MRI PDFF (Pearson correlation coefficient *R* = − 0.037, *p* = 0.89, n = 16). (**B**). BMI values were also not significantly correlated with EIT liver conductivity values (*R* =−0.19, *p* = 0.47, n = 16). (**C**) EIT liver conductivity values were negatively correlated with MRI PDFF (R = −0.69, *p* = 0,003, n = 16). The shaded areas reflect the 95% confidence intervals of the linear slope.

Using the MRI PDFF and EIT conductivity data from Table 1, we performed Pearson’s correlation analyses to determine if the BMI correlates with MRI PDFF or EIT conductivity. We observed that the correlation between BMI and MRI PDFF (*R* = − 0.037, *p* = 0.89, n = 16) or between BMI and EIT (*R* = −0.19, *p* = 0.47, n = 16) was statistically insignificant (Fig. 3A-B). However, the confidence interval plot revealed inverse correlation between EIT and MRI PDFF (*R* = −0.69, *p* = 0.003, n = 16) (Fig. 3C). This finding supports the use of EIT conductivity as an index for non-invasive detection of liver fatty infiltrate.

### Correlation analyses with the demographic and anthropometric parameters, MRI PDFF, and EIT conductivity

**Figure 4:**
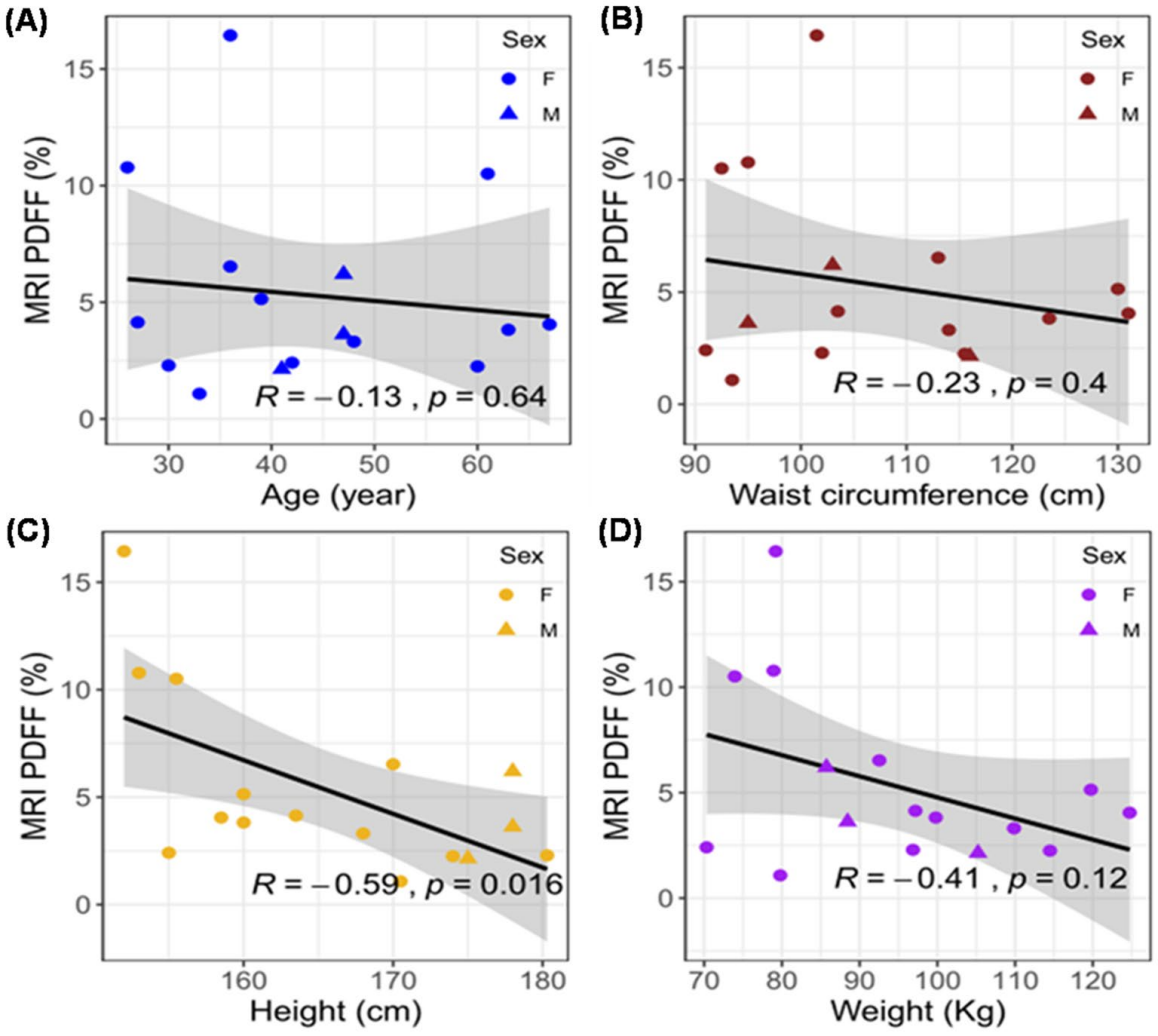
MRI PDFF vs. age, waist, height, and weight. The Pearson correlation coefficients (*R*) and *p* values were analyzed for (A) age, (B) waist circumference, (C) height, and (D) weight. The circles denote female subjects and triangles denote male subjects. The 95% confidence intervals of the linear slopes are illustrated as shaded area. *R* values are −0.13 for age (*p* = 0.64, n = 16), −0.23 for waist circumference (*p* = 0.4, n = 16), −0.59 for height (*p* = 0.016, n = 16)., and −0.41 for weight (*p* = 0.12, n = 16), demonstrating low to intermediate correlation with MRI PDFF.

**Figure 5:**
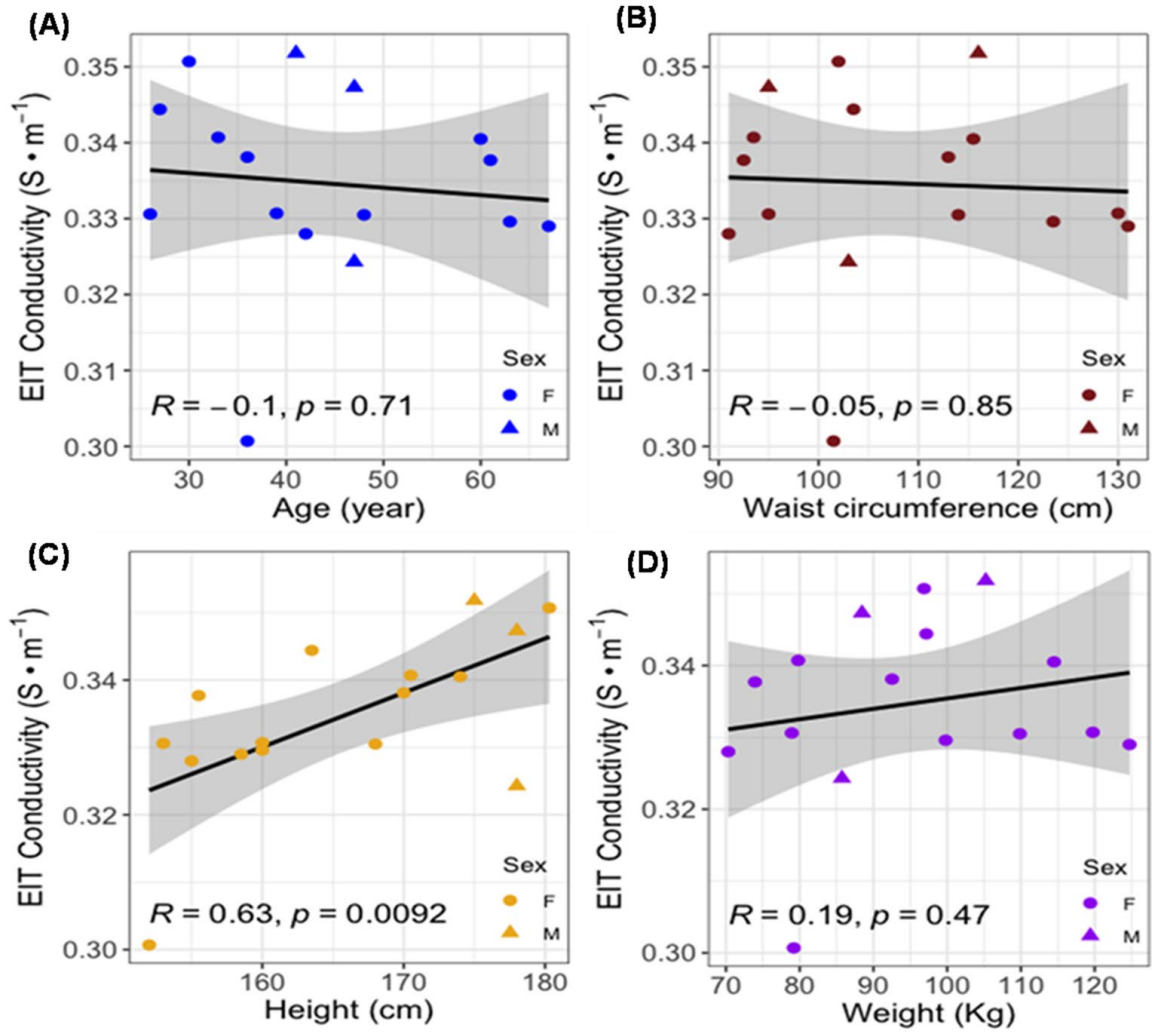
EIT liver conductivity vs. age, waist, height, and weight. The *R* values for age (R =−0.1, *p* = 0.71, n = 16), waist circumference (R =−0.05, *p* = 0.85, n = 16), height (R =0.63,* p* = 0.0092, n = 16) and weight (R = 0.19,* p* = 0.47, n = 16) demonstrate low to intermediate correlation with EIT conductivity.

To identify fatty liver infiltrate in the enrolled subjects (BMI > 25), we performed individual correlation analyses with waist circumference, height, and weight (Table 2). We compared the correlation coefficients between MRI PDFF and demographic as well as anthropometric parameters in 16 subjects (Fig. 4). Following the Bonferroni correction for multi-testing, the correlations with age (*R* = −0.13, *p* = 0.64, n = 16), waist circumference (*R* = −0.23, *p* = 0.4, n = 16), height (*R* = −0.59, *p* = 0.016, n = 16) and weight (*R* = −0.41, *p* = 0.12, n = 16) were statistically insignificant when multiple testing was considered, albeit height was significant at the nominal cut off of 0.05. We further compared the correlation coefficients between liver EIT and demographic and anthropometric parameters in 16 subjects (Fig. 5). The correlation with age (*R* = −0.1, *p* = 0.71, n = 16), waist circumference (*R* = −0.05, *p* = 0.85, n = 16), height (*R* = 0.63, *p* = 0.0092, n = 16) and weight (*R* = 0.19, *p* = 0.47, n = 16) were statistically insignificant when multiple testing was considered, and again height is nominally significant. Thus, these analyses corroborate that BMI and other parameters were not correlated with liver fat infiltrate in our overweight subjects.

**Table 2:** Demographics of overweight subjects. The demographics of 19 subjects, including sex, BMI, age, waist circumference, height, and weight, are demonstrated. (Subject 4: electrode malfunction, Subject 14: renal failure, Subject 18: leukemia, * asterisk).

Subjects	Sex	BMI(Kg·m^−2^)	Age (year)	Waist Circumference (cm)	Height (cm)	Weight (kg)
1	M	34.4	41	116	175	105.2
2	F	49.7	67	131	158.5	124.7
3	F	39.0	63	123.5	160	99.8
4*	F	33.0	35	116.5	168.9	94.1
5	F	30.6	61	92.5	155.5	73.9
6	F	36.3	27	103.5	163.5	97.2
7	F	29.3	42	91	155	70.3
8	F	37.8	60	115.5	174	114.5
9	F	32.0	36	113	170	92.5
10	F	34.3	36	101.5	152	79.2
11	M	27.9	47	95	178	88.5
12	F	46.8	39	130	160	119.8
13	F	38.9	48	114	168	109.9
14*	F	25.5	74	96	163.5	68.2
15	F	33.7	26	95	153	78.9
16	F	27.4	33	93.5	170.5	79.8
17	M	27.1	47	103	178	85.7
18*	M	46.9	57	141.5	177.5	147.7
19	F	29.8	30	102	180.3	96.9

## Discussion

Non-invasive and cost-effective monitoring of fatty liver disease remains an unmet clinical need for the early identification of cardiometabolic disorders. While liver biopsy has been performed to demonstrate non-alcoholic fatty liver disease (NAFLD), the risk of bleeding and sampling errors limit its broad application to the general population. While liver MRI is considered the non-invasive reference standard, it is costly and particularly inaccessible for underserved populations. We hereby demonstrated liver EIT as a non-invasive and portable detection method for operator-independent and cost-effective detection of liver content. Our pilot study recruited 19 adults with BMI > 25 kg·m^−2^ to undergo liver MRI scans. We performed the individual liver EIT measurements with the portable multi-electrode array, and we used the MRI-acquired a priori knowledge of the liver anatomy to solve the inverse problem for EIT reconstruction. We performed correlation analyses on liver EIT vs. MRI PDFF in relation to the individual demographics^24^. To our best knowledge, we have established a statistically significant correlation between liver EIT and MRI PDFF.

EIT has been applied to clinical medicine over the past two decades. Diagnostic EIT was developed for pulmonary function and lung capacity^22^. For instance, transthoracic impedance pneumography has been demonstrated to assess lung capacity^17,25^, and EIT was used to measure myocardial motion and blood volume for cardiac output (CO) ^26,27^. EIT has also been applied for assessing conductivity in breast and brain tissues^16^. Using the multi-electrode configuration, we obtained voltage from the abdominal surface following by injection of AC current to reconstruct the EIT conductivity distribution of the liver. However, solving the nonlinear forward and inverse models for reconstructing EIT remains a computational challenge^28–34^ as they are often ill-posed problems with solution existence, uniqueness, and instability issues^33^. The non-linear inverse model for EIT reconstruction requires a priori knowledge of the anatomic boundaries to enhance the spatial resolution for establishing the absolute conductivity value^35^. To improve the EIT reconstruction, investigators have integrated EIT with other imaging modalities, including co-registration with MRI^36–38^ and introduction of ultrasonic vibration to the target tissue in the presence of the magnetic field. This integration could generate inductive currents within the liver to enhance spatial resolution, thus, obviating the need for a priori knowledge of the liver geometry and position in the abdomen for EIT reconstruction^39^.

Alternative approaches have been applied to solve the ill-posed inverse problem for EIT reconstruction. For instance, particle swarm optimization (PSO) was applied to solve EIT as a paradigm shift from the conventional Gauss–Newton methods for rapid convergence with high spatial resolution^40,41^. Recently, convolutional neural networks (CNN) have also been applied to solve the non-linearity of the inverse problem for EIT reconstruction^42,43^. Hamilton et al. obtained the absolute EIT images by combining the D-bar method with subsequent processing using the CNN technique for sharpening the EIT reconstruction^42^. Li et al. utilized deep neural networks (DNN) to directly obtain a nonlinear relationship between the one-dimensional boundary voltage and the internal conductivity ^43^. The accuracy of EIT reconstruction may be improved by employing multiple levels of the electrode arrays to circumferentially wrap around the upper abdomen. This multi-level electrode array would enable current injection and voltage recording from the entire liver for 3-D EIT reconstruction.

As a corollary, we compared the liver anatomy with MRI PDFF from a representative 3-D rendering (Fig S1A-B). The 3-D EIT conductivity distribution was reconstructed with the aid of the MRI multi-echo sequence acquired a priori knowledge (Fig S1C). The high-fat region in the MRI PDFF (red dashed box) was also detected by the EIT with the reduced conductivity. The 3-D EIT conductivity distribution reveals the inhomogeneous fat distribution as supported by the MRI 3-D rendering images (Fig S1C). With additional scanning along the z-direction, a precise conductivity distribution could be achieved to reveal the details of the heterogeneous fat distribution.

While MRI images provided the a priori knowledge to solve the ill-posed inverse problem for EIT reconstruction, alternative methods to provide the boundary conditions would allow for low-cost liver EIT screening for the underserved populations. The previous studies have proposed the sensors to integrate the detection of the EIT signals with stretch or acceleration to reconstruct the anatomical contour of the upper abdomen^44–47^. Khor et al*.* have demonstrated the wearable sensors integrating with strain gauge and EIT electrodes for measuring anatomical contour needed to monitor the neonatal lung function^45^. de Gelidi et al*.* integrated the accelerometer to detect the dorsal shape with EIT sensors for improving lung function monitoring^46^. Moreover, Darma et al*.* have combined EIT sensors with flexible stretch sensors to measure the contour of the arm for EIT reconstruction^47^. These proposed sensors have the capacity to acquire the change in resistance and to extract the curvature from each sensor based on the pre-established curvature-resistance relation. Thus, these studies provided the potential solutions for simultaneously acquiring both abdominal contours and voltage signals for EIT constructions.

A potential alternative method which is still under investigation to acquire the peripheral boundary is frequency-differential electrical impedance tomography (fdEIT). fdEIT has been proposed to address technical difficulties encountered by unknown boundary geometry and uncertainty in electrode positions from a conventional EIT imaging method^48^. fdEIT allows for reconstructing various tissue conductivity by injecting current at two distinct frequencies to the abdomen, followed by acquiring the resulting surface-voltage. Sun et al*.* have applied fdEIT to reconstruct the conductivity distribution of calf muscle in response to stimulation. Their reconstruction images illustrated the potential feasibility of distinguishing the boundary between the muscle and subcutaneous fat in human calf^49^. In addition, Menden et al*.* have recently proposed a reconstruction algorithm for frequency-differential EIT using absolute values^50^. The preliminary result demonstrated the potential for differentiating organ and spine boundaries. Moreover, Yao et al*.* have demonstrated the detection of multicomponent distribution through fdEIT^51^. Hence, accurately selecting the two frequencies would have potential to acquire the peripheral boundary and differentiate the fatty from the non-fatty tissues by virtue of tissue-specific electrical properties (Table S1)^48–50^. As a result, the peripheral layer can be used as the a priori knowledge to solve the inverse problem for EIT reconstruction, thus, obviating the need for MRI. Furthermore, establishing an atlas of external liver MRI images and an anthropometric database would help calibrate the boundary conditions of the liver to improve EIT reconstruction.

Our liver EIT results further reveal the effect of fluid accumulation on the liver EIT conductivity. If we included two subjects with electrolytes abnormities (leukemia and renal failure), the correlation value between EIT and MRI PDFF was decreased from R = −0.69 (p = 0.003, n = 16) to R = −0.21 (p = 0.4, n = 18) (Fig S2A). If we further excluded the two subjects with anemia, the correlation improved from R = −0.69 (p = 0,003, n = 16) to R = −0.70 (p = 0.0049, n = 14) (Fig S2B). In this case, the pre-existing medical conditions, including leukemia, renal failure, and anemia, disrupted the impedimetric property of liver, resulting in altered EIT conductivity.

In summary, we enrolled overweight subjects to undergo MRI scans and liver EIT measurements to reconstruct the EIT conductivity distribution. We demonstrated that the increase in liver EIT conductivity is correlated with a decrease in MRI PDFF. As a corollary, the 3-D EIT conductivity map revealed the heterogeneous distribution of fatty gradient as evidenced by the 3-D MRI PDFF. Our correlation analyses supported that subject-specific EIT offers a non-invasive and portable method for the operator-independent and cost-effective detection of hepatic fat infiltrate in the overweight populations.

## Methods

### Study design

**Figure 6:**
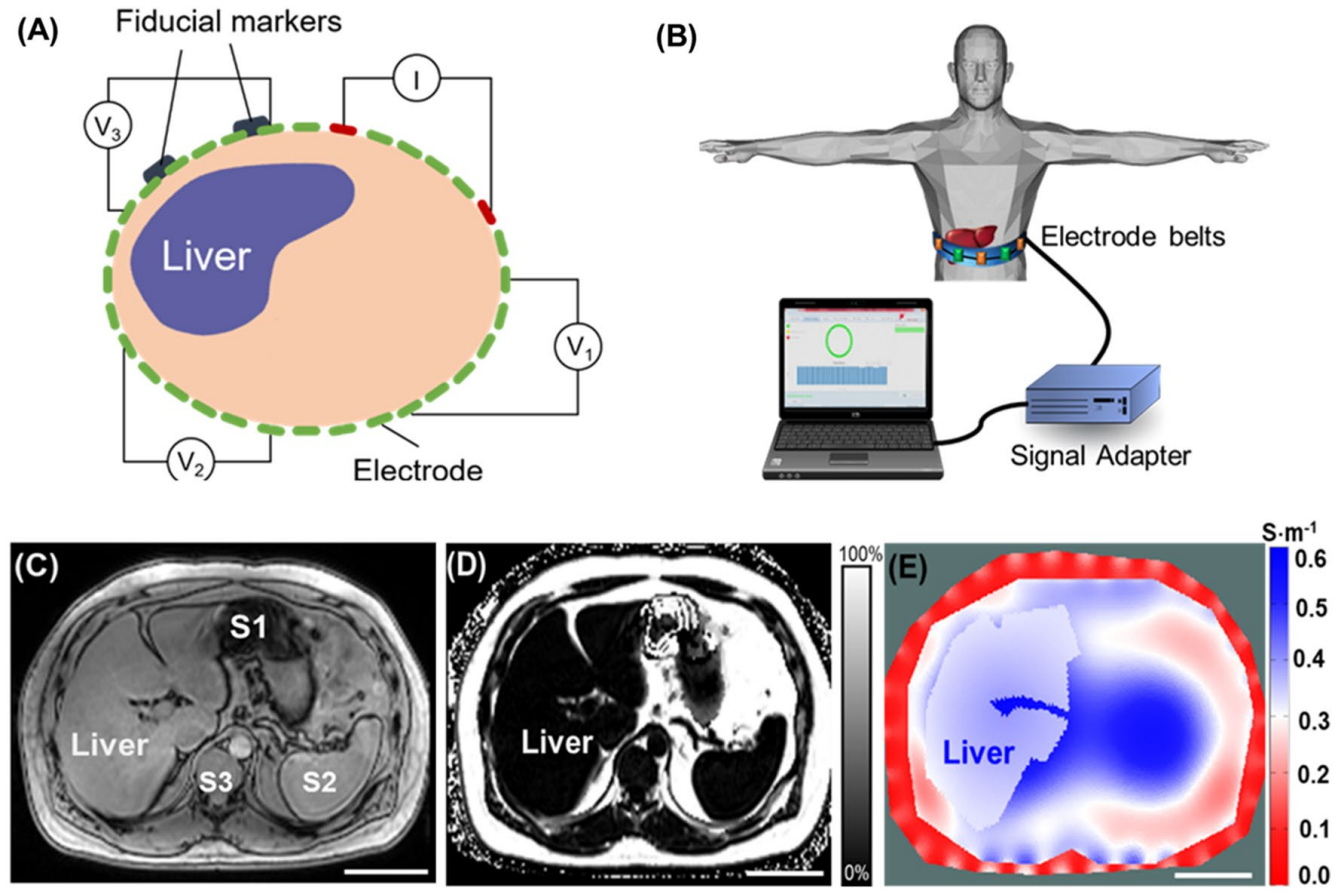
Schematic of EIT measurement, reconstruction, and 2-D representation. (**A**) Schematic illustrates circumferential electrode placement around the abdomen for pairwise voltage measurements. The fiducial markers indicate the anatomic level at which the multi-electrode array was circumferentially positioned for liver EIT measurements. (**B**) Thirty-two electrodes were adhered to the abdomen, as indicated by the fiducial markers. The recorded voltage signals were input to a signal adaptor and the data acquisition channels for EIT measurements. (**C**) A representative MRI multi-echo image demarcates the boundary conditions for the abdomen, liver, stomach, and spleen. S1: Stomach, S2: Spleen, S3: Spine. (**D**) A representative PDFF map is compared with the corresponding EIT image. (**E**) A representative 2-D EIT image reveals the conductivity distribution. Scale bar: 8 cm.

The recruitment of human subjects was conducted at the UCLA Center for Human Nutrition in compliance with the UCLA Human Subjects Protection Committee. The study protocol (#15–001,756) was approved by the UCLA Internal Review Board. All subjects provided written informed consent before participating in research procedures. All experiments were performed in accordance with relevant named guidelines and regulations. We enrolled a total of 19 volunteers including, 15 females and 4 males, from 27 to 74 years old with a waist circumference from 91 cm to 141.5 cm and body mass index (BMI) defined as body mass divided by the square of the body height from 25.5 to 46.8 kg/m^2^ (Fig. 1). Inclusion criteria for all subjects included the ability to travel for phlebotomy for whole blood collection, no prescription or over-the-counter medications for weight loss, and absence of alcohol consumption, no weight change > 5 pounds in the previous 3 months, overweight with BMI > 25, and waist circumference > 40″ for men or > 35″ for women. All subjects must be able to follow instructions and to consent. Exclusion criteria for all subjects included coronary artery disease on medications, claustrophobia, previous liver cancer, liver surgery, alcoholism (DSM-5 criteria: alcohol abuse or dependence), metallic implants or other factors hazardous to the MRI scanner as per the MRI safety guidelines, and body weight > 300 pounds (weight and size restrictions for undergoing MRI). Note that an MRI scan was performed to establish PDFF for quantifying fatty infiltrates in the liver. Clinical demographic and physical characteristics of human subjects were collected in terms of gender, BMI (kg·m^−2^), age (years), waist circumference (cm), height (cm), and weight (kg) (Table 2). Following enrollment and consent, the subjects underwent a 30-min liver MRI scan, including multi-echo imaging for mapping the proton density fat fraction (PDFF) (Fig. 6). Next, EIT measurement was acquired by placing 32 electrodes to the upper abdominal region, as indicated by the fiduciary markers immediately following the MRI scan (Fig. 6A). A pair of electrodes was used to inject the AC current to the abdomen, and the electrode array was used to record voltage by the pairwise algorithm (Fig. 6B). Liver MRI provided the a priori knowledge of the boundary conditions needed for the EIT conductivity map reconstruction and PDFF (Fig. 6C-D). EIT conductivity map was reconstructed to distinguish the liver conductivity gradient from other tissues or organs (Fig. 6E). Finally, subject-specific EIT (conductivity map) was compared with the corresponding MRI PDFF (Fig. 6E).

### Determination of Liver MRI proton-density fat fraction PDFF

Non-contrast-enhanced abdominal MRI scans were performed on a 3-Tesla system (Skyra or Prisma, Siemens, Erlangen, Germany) using a body array and a spine array coils. The protocol included breath-held anatomical scouts, a breath-held T_2_-weighted 2-D multi-slice half-Fourier single-shot turbo spin-echo (HASTE) sequence, and a breath-held 3-D multi-echo gradient-echo sequence (TE = 1.23, 2.46, 3.69, 4.92, 6.15, 7.38 ms; TR = 8.94 ms, flip angle = 4 deg, typical field of view = 400 × 350 × 256 mm^3^, typical matrix size = 192 × 168 × 64, parallel imaging factor = 4, typical scan time = 19 s) to quantify PDFF. Scanner software (LiverLab, Siemens, Erlangen, Germany), which utilized a multi-peak fat spectral model with single R_2_* for multi-step signal fitting, was used to calculate PDFF^52^. The MRI images and PDFF maps were saved in DICOM format and downloaded from the scanner for analysis.

To ensure alignment of the subsequent EIT slice position to a corresponding mid-liver MRI slice, we affixed two to three MRI-visible fiducial markers (MR-SPOT 122, Beekley Medical, Bristol, CT) to the skin above the expected mid-liver region prior to performing the MRI scan (Fig. 6A). The positioning of the fiducial markers was examined on the anatomical scouts. If needed, the MRI technologist would re-position the fiducial markers on the subject’s abdomen and re-acquire the scouts. At least one adjustment would be required, and this entire alignment required less than 3 min.

The echo 1 (TE = 1.23) magnitude images from the 3-D multi-echo gradient-echo sequence were used for contouring the body and the liver to create a 3-D anatomy model. An axial slice in the MRI PDFF maps that contained MRI-visible fiducial markers was selected for analysis. Five circular regions of interest (ROIs) with an area of 5 mm^2^ were delineated in the slice with fiducial markers by a trained researcher to avoid blood vessels, bile ducts, and imaging artifacts, and at least 1–2 cm away from the liver capsule. The mean PDFF from the ROIs (0–100%) was reported for each subject.

### Theoretical Framework for EIT reconstruction (EIDORS)

The EIT imaging reconstruction was implemented as previously described^11^. Following the injection of a known current to the abdomen, an EIT conductivity map across the abdomen was reconstructed with a set of voltages recorded by an electrode array placed on the surface of the upper abdomen (see Fig S3)^7^. With a priori knowledge of the target object (liver), the geometric boundary conditions were established with a high degree of precision to mitigate instability inherent from the ill-posed EIT inverse problem^53^(Fig. 6D), and the solution was obtained by using a regularized Gauss–Newton (GN) type solver(Fig. S3).

The Gauss–Newton (GN) type solver calculates the conductivity by minimizing $\emptyset$, the L2 norm (the square root of the sum of the squares of the values) of the difference between the measured voltage ${V}_{o},$ and a function of the conductivity $f(\sigma )$:

1 $$\emptyset ={\Vert {V}_{o}-f(\sigma )\Vert }$$

where $f(\sigma )$ is considered to be the "forward problem" derived from the Laplace equations:

2 $$\nabla \cdot \left(-\sigma \nabla V\right)=0$$

By taking the first-order Taylor series expansion of $\emptyset$:

3 $$\emptyset ={\Vert {V}_{o}-f(\sigma )\Vert }\cong {\Vert \left({V}_{o}-f\left({\sigma }_{0}\right)\right)-J(\sigma -{\sigma }_{0})\Vert }$$

where ${\sigma }_{0}$ is a reference conductivity value, and $J$ is the Jacobian matrix of our inverse problem.

By setting $\frac{\partial {\emptyset }}{\partial\upsigma }=0$, we minimized $\emptyset$ and obtained $\sigma$ as follows:

4 $$\sigma ={\sigma }_{0}+{\left({J}^{T}J\right)}^{-1}{J}^{T}({V}_{o}-f\left({\sigma }_{0}\right))$$

Equation (4) is an unconstrained GN form of the inverse problem. Due to the ill-posed nature of the EIT inverse problem, achieving a converged solution from this unconstrained GN form is challenging. The solution $\sigma$ is highly sensitive to perturbations in voltage ($V$) measurement, which means a small noise in $V$ leads to instability in the final solution. A general method to mitigate the issue is to introduce a constraint term that sways the solution towards the preferred solution:

5 $$\emptyset^{2}={\Vert \varepsilon \Vert }^{2}+\lambda {\Vert \Gamma \sigma \Vert }^{2}$$

To balance the tradeoff between fitting the error and constraining the solution from the undesired properties, we incorporated a constraint term, $\lambda {\Vert \Gamma \sigma \Vert }^{2},$ to the objective function and the resulted form is commonly known as the Tikhonov Regularization. The coefficient, $\lambda$, is the regularization parameter that suppresses the conductivity spikes in the solution space.

With a priori conductivity within a similar area, the term, $\Gamma$, was introduced as a “weighted” Laplacian operator that enables us to adjust more properties of the conductivity and suppress the non-smooth regions. Akin to the present work, this strategy is useful in medical imaging, where a priori anatomic information of individual organs was obtained from MRI multi-echo sequence and integrated with the EIT solutions. By applying the regulation term to Eq. (2), we generated the solution as follows:

6 $${\sigma }_{1}={\sigma }_{0}+{\left({J}^{T}J+\lambda {\Gamma }^{T}\Gamma \right)}^{-1}{J}^{T}(V-f\left({\sigma }_{0}\right))$$

To obtain the absolute conductivity mapping, we adopted an iterated approach by first assuming an arbitrary conductivity, ${\sigma }_{0}$, which is used to calculate $J$, $\Gamma$, and $f\left({\sigma }_{0}\right)$. From Eq. (6), we calculated a new conductivity value set, ${\sigma }_{1}$ to generate a new set of $J$, $\Gamma$ and $f\left({\sigma }_{1}\right)$. The iteration continued until the difference between ${\sigma }_{n}$ and ${\sigma }_{n-1}$ reached a minimally desired value.

In this study, we adopted the online open-source software suite EIDORS (version 3.8) for EIT image reconstruction. An inverse finite element model was aided with a mesh generator (Netgen) for the reconstruction of liver EIT image. Rather than using a presumed geometry for the finite element model, we combined the MRI multi-echo images-acquired geometric information with the multi-electrode-measured voltage data to reconstruct the liver EIT conductivity map. As a result, the computational errors from the variations in the geometry of the abdomen of different subjects were reduced.

### EIT measurement and reconstruction for fat infiltrate

Following MRI scans, the subjects underwent EIT measurement. The MRI-visible fiducial markers on the abdomen facilitated the circumferential positioning of EIT electrodes. Next, the subjects were instructed to be in the supine position, and to perform breath-holds (e.g., end inspiration) as they did for the MRI scan. This instruction ensured that the EIT slice matched with the level of the mid-liver MRI slice. Electrical measurement and data acquisition were conducted using the Swisstom EIT Pioneer Set (Swisstom AG, Switzerland). An array of disposable surface electrocardiogram electrodes (Covidien, Ireland) was attached to the skin of the subject, and each of them was connected to one of the 32 data acquisition channels of the Swisstom system, which was interfaced with the controlling computer via a separate module (Fig. 6A,B). The AC currents with programmable magnitude from 1–4 mA were injected to the upper abdomen at 50 kHz and 250 kHz respectively through the selected channels, and the resulting voltage responses were recorded by a separate pair of electrodes. A “skipping 4” pattern was used for current injection and voltage recording^54^ (Fig. 6A). Based on the anatomy of the liver, we established both 2-D and 3-D forward models for EIT reconstruction using the EIDORS library. The acquired voltage data at 50 kHz were used to calculate 2-D and 3-D conductivity distribution. Following EIT reconstruction of the liver conductivity maps, we compared the liver conductivity (S m^−1^) and MRI PDFF with the subject-specific demographics, and we generated the confidence interval plots to demonstrate the correlation between liver EIT conductivity and MRI PDFF.

### Statistical analysis

All statistical analyses were conducted using R. We performed individual correlation analyses of EIT and MRI-PDFF with subjects’ demographics after excluding individuals with known disorders such as chronic lymphocytic leukemia and renal failure. We also excluded one liver EIT measurement due to electrode malfunction for a final sample size of n = 16 for these demographic comparisons (Fig. S4). To assess how the preexisting medical conditions affect the correlation between EIT and MRI-PDFF, we further calculated the correlations with (n = 18) and without individuals with pre-existing medical conditions that could disturb the circulating and tissue electrolytes (n = 14) (Fig. S4). The correlation between liver conductivity and MRI PDFF and the correlations with demographic variables were assessed by Pearson’s correlation analysis, and the significance threshold was adjusted using a Bonferroni correction for multiple testing. In the associated scatter plots, we also included the 95% confidence intervals.

## Supplementary Information

Supplementary Information.

## Data Availability

The authors declare that the main data supporting the findings of this study are available within the article and its Supporting Information files. Extra data are available from the corresponding author on a reasonable request.

## References

[1] Ahlqvist E (2018). Novel subgroups of adult-onset diabetes and their association with outcomes: A data-driven cluster analysis of six variables. Lancet Diab. Endocrinol..

[2] Lazo M, Clark JM (2008). The epidemiology of nonalcoholic fatty liver disease: A global perspective. Semin. Liver Dis..

[3] Kemmer N (2013). Nonalcoholic fatty liver disease epidemic and its implications for liver transplantation. Transplantation.

[4] Marchesini G (2003). Nonalcoholic fatty liver, steatohepatitis, and the metabolic syndrome. Hepatology.

[5] Vernon G, Baranova A, Younossi ZM (2011). Systematic review: the epidemiology and natural history of non-alcoholic fatty liver disease and non-alcoholic steatohepatitis in adults. Aliment. Pharmacol. Ther..

[6] Bravo AA, Sheth SG, Chopra S (2001). Liver biopsy. N. Engl. J. Med..

[7] Reeder SB, Hu HH, Sirlin CB (2012). Proton density fat-fraction: a standardized MR-based biomarker of tissue fat concentration. J. Magn. Reson. Imaging.

[8] Yokoo T (2017). Linearity, bias, and precision of hepatic proton density fat fraction measurements by using MR imaging: A meta-analysis. Radiology.

[9] Shuster A, Patlas M, Pinthus J, Mourtzakis M (2012). The clinical importance of visceral adiposity: A critical review of methods for visceral adipose tissue analysis. Br. J. Radiol..

[10] Tang A, Cloutier G, Szeverenyi NM, Sirlin CB (2015). Ultrasound elastography and MR elastography for assessing liver fibrosis: Part 1, principles and techniques. Am. J. Roentgenol..

[11] Luo Y (2018). Non-invasive electrical impedance tomography for multi-scale detection of liver fat content. Theranostics.

[12] Brown BH (2003). Electrical impedance tomography (EIT): A review. J. Med. Eng. Technol..

[13] Cheney M, Isaacson D, Newell JC (1999). Electrical impedance tomography.. SIAM Rev..

[14] Christ, M., Kenig, C. E. & Sadosky, C. *Harmonic analysis and partial differential equations: essays in honor of Alberto P. Calderón*. (University of Chicago Press, 2001).

[15] Holder, D. S. *Electrical impedance tomography: methods, history and applications*. (CRC Press, 2004).

[16] Bayford RH (2006). Bioimpedance tomography (electrical impedance tomography). Annu. Rev. Biomed. Eng..

[17] Wilkinson J, Thanawala V (2009). Thoracic impedance monitoring of respiratory rate during sedation–is it safe?. Anaesthesia.

[18] Frerichs I, Becher T, Weiler N (2014). Electrical impedance tomography imaging of the cardiopulmonary system. Curr. Opin. Crit. Care.

[19] Nguyen, D. M., Andersen, T., Qian, P., Barry, T. & McEwan, A. Electrical Impedance Tomography for monitoring cardiac radiofrequency ablation: a scoping review of an emerging technology. *Med. Eng. Phys.* (2020). 10.1016/j.medengphy.2020.07.025 32977921

[20] Isaacson D, Cheney M, Newell JC (1992). Comments on reconstruction algorithms. Clin. Phys. Physiol. Meas..

[21] Packard RRS (2017). 3-D electrochemical impedance spectroscopy mapping of arteries to detect metabolically active but angiographically invisible atherosclerotic lesions. Theranostics.

[22] Brown BH, Seagar AD (1987). The Sheffield data collection system. Clin. Phys. Physiol. Meas..

[23] Bachtiar, V. *et al.* Repeatability and reproducibility of multiparametric magnetic resonance imaging of the liver. *PloS one* **14**, e0214921 (2019). 10.1371/journal.pone.0214921 PMC6457552 30970039

[24] Hu HH, Li Y, Nagy TR, Goran MI, Nayak KS (2011). Quantification of absolute fat mass by magnetic resonance imaging: a validation study against chemical analysis. Int. J. Body Compos. Res..

[25] Brown, B. H. Impedance pneumography. (1997).

[26] Pikkemaat R, Lundin S, Stenqvist O, Hilgers R-D, Leonhardt S (2014). Recent advances in and limitations of cardiac output monitoring by means of electrical impedance tomography. Anesth. Analg..

[27] Zlochiver S, Freimark D, Arad M, Adunsky A, Abboud S (2006). Parametric EIT for monitoring cardiac stroke volume. Physiol. Meas..

[28] Krautblatter, M., Hauck, C. (2007) Electrical resistivity tomography monitoring of permafrost in solid rock walls. *J. Geophys. Res. Earth Surface* **112**, 1.

[29] Bolton GT (2007). Development of an electrical tomographic system for operation in a remote, acidic and radioactive environment. Chem. Eng. J..

[30] Rücker C, Günther T, Spitzer K (2006). Three-dimensional modelling and inversion of DC resistivity data incorporating topography—I Modelling. Geophys. J. Int..

[31] Heinrich S, Schiffmann H, Frerichs A, Klockgether-Radke A, Frerichs I (2006). Body and head position effects on regional lung ventilation in infants: An electrical impedance tomography study. Intens. Care Med..

[32] Adler A (2012). Whither lung EIT: Where are we, where do we want to go and what do we need to get there?. Physiol. Meas..

[33] Calderón AP (2006). On an inverse boundary value problem. Comput. Appl. Math..

[34] Cheney M, Isaacson D, Newell JC (1999). Electrical impedance tomography. SIAM Rev..

[35] Seo, J. K. & Woo, E. J. *Nonlinear inverse problems in imaging*. (John Wiley & Sons, 2012).

[36] Crabb M (2014). Mutual information as a measure of image quality for 3D dynamic lung imaging with EIT. Physiol. Meas..

[37] Baek KI (2018). Advanced microscopy to elucidate cardiovascular injury and regeneration: 4D light-sheet imaging. Prog. Biophys. Mol. Biol..

[38] Chang C-C (2021). Three-dimensional imaging coupled with topological quantification uncovers retinal vascular plexuses undergoing obliteration. Theranostics.

[39] Grasland-Mongrain P, Mari J-M, Chapelon J-Y, Lafon C (2013). Lorentz force electrical impedance tomography. Irbm.

[40] Chen, M.-Y., Hu, G., He, W., Yang, Y.-L. & Zhai, J.-Q. in *Life system modeling and intelligent computing* 342–350 (Springer, 2010).

[41] Feitosa, A. R., Ribeiro, R. R., Barbosa, V. A., de Souza, R. E. & dos Santos, W. P. in *5th ISSNIP-IEEE Biosignals and Biorobotics Conference (2014): Biosignals and Robotics for Better and Safer Living (BRC).* 1–6 (IEEE).

[42] Hamilton SJ, Hauptmann A (2018). Deep D-bar: Real-time electrical impedance tomography imaging with deep neural networks. IEEE Trans. Med. Imaging.

[43] Li X (2019). A novel deep neural network method for electrical impedance tomography. Trans. Inst. Meas. Control..

[44] Luo, Z., Li, J., Hong, G. & Li, H. Strain‐based displacement field reconstruction method for thin rectangular plate through orthogonal deflection curves bridging. *Structural Control and Health Monitoring* **27**, e2457 (2020).

[45] Khor JM, Tizzard A, Demosthenous A, Bayford R (2014). Wearable sensors for patient-specific boundary shape estimation to improve the forward model for electrical impedance tomography (EIT) of neonatal lung function. Physiol. Meas..

[46] de Gelidi, S. *et al.* Torso shape detection to improve lung monitoring. *Physiological measurement* **39**, 074001 (2018). 10.1088/1361-6579/aacc1c 29894309

[47] Darma P, Baidillah M, Sifuna M, Takei M (2020). Real-time dynamic imaging method for flexible boundary sensor in wearable electrical impedance tomography. IEEE Sens. J..

[48] Seo JK, Lee J, Kim SW, Zribi H, Woo EJ (2008). Frequency-difference electrical impedance tomography (fdEIT): algorithm development and feasibility study. Physiol. Meas..

[49] Sun, B. *et al.* Evaluation of the effectiveness of electrical muscle stimulation on human calf muscles via frequency difference electrical impedance tomography. *Physiological Measurement* **42**, 035008 (2021). 10.1088/1361-6579/abe9ff 33631732

[50] Menden, T. *et al.* Reconstruction algorithm for frequency-differential EIT using absolute values. *Physiological measurement* **40**, 034008 (2019). 10.1088/1361-6579/ab0b55 30818291

[51] Yao, W. *et al.* in *Twelfth International Conference on Graphics and Image Processing (ICGIP 2020).* 117200B (International Society for Optics and Photonics).

[52] Zhong X (2014). Liver fat quantification using a multi-step adaptive fitting approach with multi-echo GRE imaging. Magn. Reson. Med..

[53] Ider YZ, Birgül Ö (2000). Use of the magnetic field generated by the internal distribution of injected currents for electrical impedance tomography (MR-EIT). Turk. J. Electr. Eng. Comput. Sci..

[54] Adler A, Gaggero PO, Maimaitijiang Y (2011). Adjacent stimulation and measurement patterns considered harmful. Physiol. Meas..

