# Supplementary material for: Electrical impedance tomography for non-invasive identification of fatty liver infiltrate in overweight individuals
Source: Sci Rep. 2021 Oct 6;11:19859. doi: 10.1038/s41598-021-99132-z (PMC8494919; doi:10.1038/s41598-021-99132-z)
Supplement: Supplementary file 1 — Supplementary Information. [file 41598_2021_99132_MOESM1_ESM.pdf]

Supplementary Information

Electrical Impedance Tomography for Non-Invasive Identification of Fatty Liver Infiltrate in Overweight Individuals

Chih-Chiang Chang[1,4+], Zi-Yu Huang[2+], Shu-Fu Shih[1,3], Yuan Luo[2], Arthur Ko[4], Qingyu Cui[4], Jennifer Sumner[5], Susana Cavallero[4], Swarna Das[1], Wei Gao[2], Janet Sinsheimer[6,7, 8], Alex Bui[1,3], Jonathan P. Jacobs[4,9,10], Päivi Pajukanta[7,11], Holden Wu[1,3], Yu-Chong Tai[2], Zhaoping Li[4,10,12], and Tzung K. Hsiai[1,2,4,10*]

[1]Department of Bioengineering, University of California, Los Angeles, Los Angeles, CA
[2]Department of Medical Engineering, California Institute of Technology, Pasadena, CA
[3]Department of Radiological Sciences, David Geffen School of Medicine at UCLA, Los Angeles, CA
[4]Department of Medicine, David Geffen School of Medicine at UCLA, Los Angeles, CA
[5]Department of Psychology, College of Life Sciences, UCLA, Los Angeles, CA
[6]Department of Biostatistics, Fielding School of Public Health, UCLA, Los Angeles, CA
[7]Department of Human Genetics, David Geffen School of Medicine at UCLA, Los Angeles, CA
[8]Computational Medicine, David Geffen School of Medicine at UCLA, Los Angeles, CA
[9]Division of Digestive Diseases, David Geffen School of Medicine at UCLA, Los Angeles, CA
[10]Greater Los Angeles VA Healthcare System, Los Angeles, CA
[11]Institute for Precision Health, David Geffen School of Medicine at UCLA, Los Angeles, CA
[12]Center for Human Nutrition, David Geffen School of Medicine at UCLA, Los Angeles, CA

+Both authors contributed equally.

Supplementary Materials

Fig. S1. 3-D MRI PDFF mapping vs. 3-D EIT image.

Fig. S2. Sub-analysis of EIT liver conductivity vs. MRI PDFF for all subjects and additional exclusion of anemic subjects.

Fig. S3. Schematic flow of EIT reconstruction.

Fig. S4. Subject recruitment flow chart.

Table S1. Conductivities of human tissue.

Supplementary Figure 1

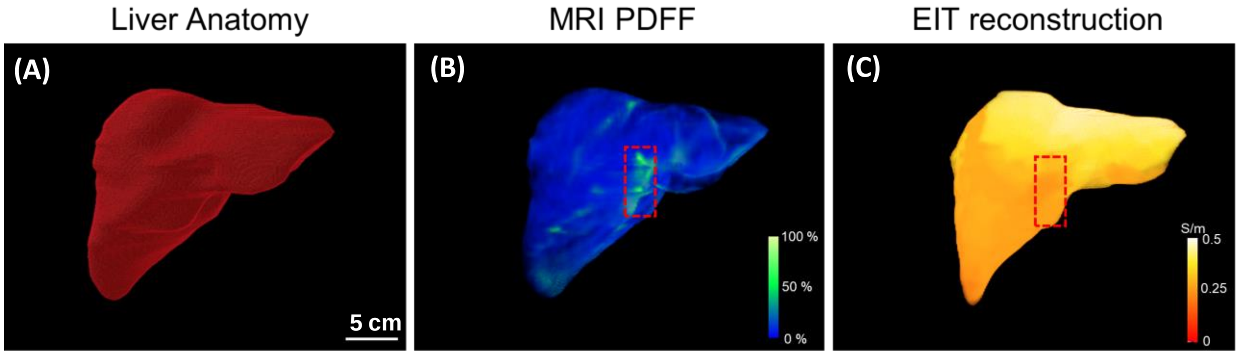

Supplementary Figure 1. 3-D MRI PDFF mapping vs. 3-D EIT image. (A) The representative 3-D liver boundary condition was established following segmentation of the MRI multi-echo imaging. **(B)** 3-D MRI PDFF mapping reveals a heterogeneous distribution of MRI PDFF. The red dashed box highlights the region with a relatively high fat fraction. **(C)** 3-D EIT image unveils the heterogeneous gradient of conductivity. The dash red box is consistent with that of MRI PDFF mapping. Thus, the 3-D comparison between MRI multi-echo imaging and EIT image further supports the correlation between MRI fat fraction and EIT conductivity. Scale bar: 5 cm.

Supplementary Figure 2

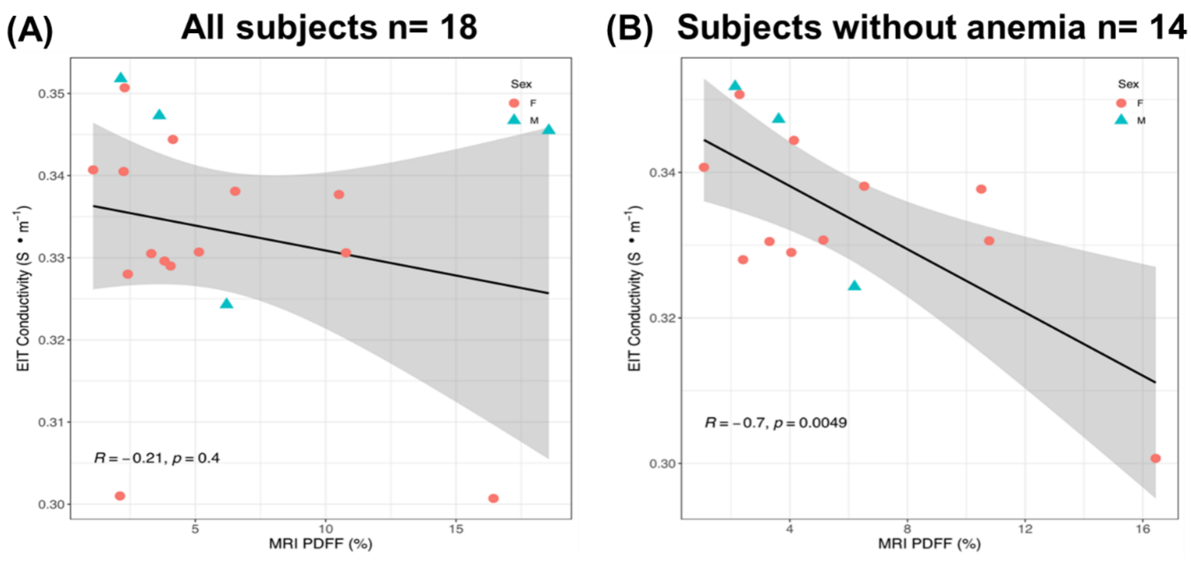

Supplementary Figure 2. Sub-analysis of EIT liver conductivity vs. MRI PDFF for all subjects and additional exclusion of anemic subjects. (A) The negative correlation between EIT conductivity and MRI PDFF was reduced to R=-0.21 in the presence of preexisting medical conditions implicated in disturbing tissue electrolytes (p = 0.4, n = 18). **(B)** The correlation between EIT liver vs. MRI PDFF was increased to R = -0.70 in the absence of anemia subjects (p = 0.0049, n= 14). The shaded areas reflect the 95% confidence intervals of the linear slopes.

Supplementary Figure 3

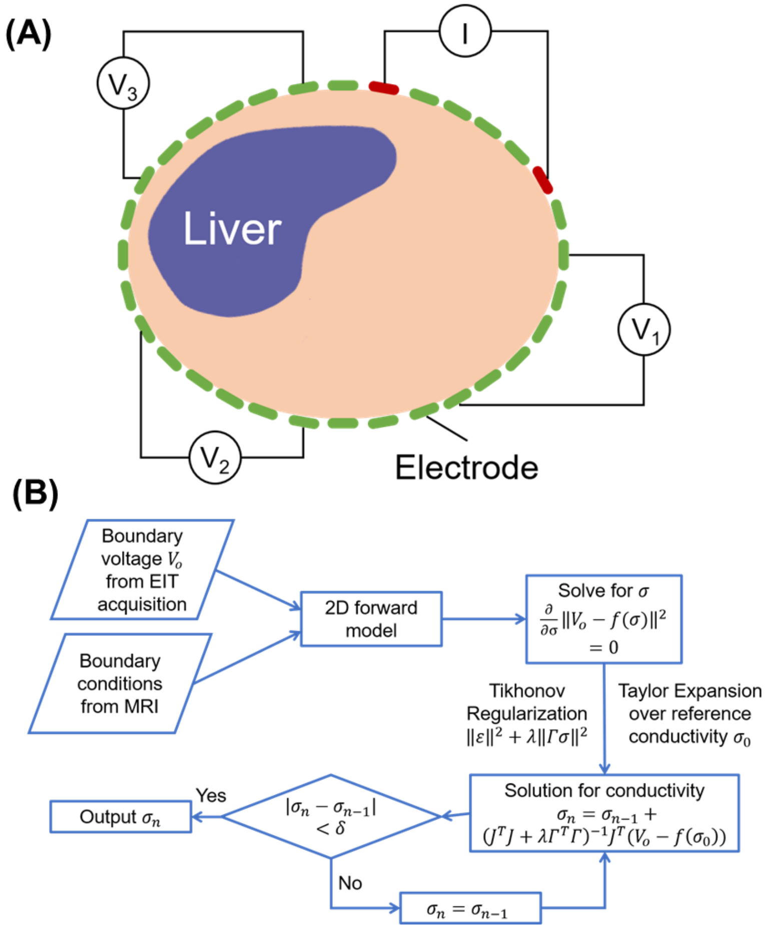

Supplementary Figure 3. Schematic flow of EIT reconstruction. (A) "Skipping 4" pattern was used for both current injection and voltage acquisition. There were 4 electrodes separating each pair of stimulating and detecting electrodes. **(B)** EIT reconstruction was established by solving the inverse problem via a regularized Gauss-Newton (GN) type solver.

Supplementary Figure 4

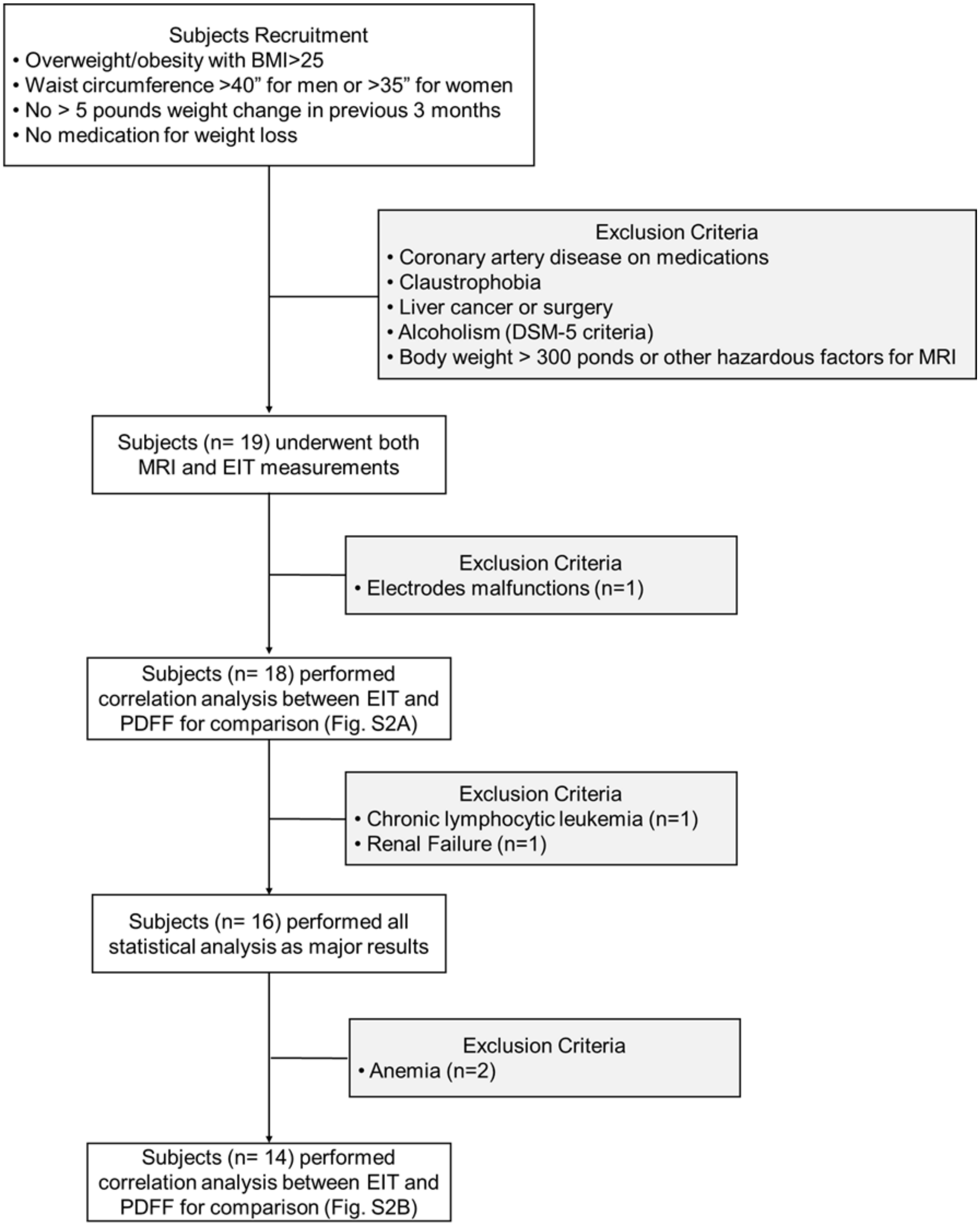

Supplementary Figure 4. Subject recruitment flow chart.

Tissue	$S \cdot m^{-1}$	Tissue	$S \cdot m^{-1}$
liver	0.07	fat	0.04
lung	0.14	musle	0.35
heart	0.10	bone marrow	0.06
kidney	0.10	skin	0.10
intestine	0.35	blood	0.70
stomach	0.50	cartilage	0.18

Supplementary Table 1 Conductivities of human tissues at 50 kHz [$S \cdot m^{-1}$].
